# Sex ratio shift after frozen single blastocyst transfer in relation to blastocyst morphology parameters

**DOI:** 10.1038/s41598-024-59939-y

**Published:** 2024-04-25

**Authors:** Tiantian Wang, Lixia Zhu, Mingru Yin, Weina Yu, Jing Dong, Wei Jin, Qifeng Lyu, Lei Jin, Hui Long

**Affiliations:** 1 Department of Assisted Reproduction, Shanghai Ninth People’s Hospital, Shanghai Jiao Tong University School of Medicine, Shanghai, 200011 China; 2 Reproductive Medicine Center, Tongji Hospital, Tongji Medical College, Huazhong University of Science and Technology, Wuhan, 300559 China

**Keywords:** Sex ratio, Blastocyst transfer, Morphology, Trophectoderm, Inner cell mass, Expansion degree, Developmental biology, Medical research

## Abstract

The sex ratio shift was observed in peoples who underwent ART treatment. Moreover, there is limited evidence on differences in sex ratio between single frozen-thawed blastocyst morphology, insemination type and transfer days. So further research is needed in this area with regard to factors possibly affecting the sex ratio. Retrospective study based on multicenter including two large assisted reproduction centers in Shanghai and Wuhan in China. A total of 6361 singleton delivery offspring after frozen-thawed blastocyst transfer. Propensity score weighting and logistic regression models were used to estimate the associations between blastocyst morphology grading and child sex ratio. The main outcome measures is singleton sex ratio. In our study, the primary outcome measure was sex ratio which was calculated as the proportion of male newborns among all live births. Higher quality blastocysts resulted in a higher sex ratio than single poor-quality frozen-thawed blastocyst transfer. Among the three blastocyst morphological parameters of trophectoderm (TE), Grade A and B were significantly associated with a higher sex ratio than Grade C. The similar trend was observed in both IVF and ICSI treated subgroups. As compared with expansion (4 + 3), expansion degree 6 achieved a higher sex ratio in overall populations and IVF treated subgroup. Transferring blastocysts of day 6 had the highest sex ratio both in IVF group and ICSI group. A 6.95% higher sex ratio in transferring blastocysts of day 5 in IVF group than those in ICSI group. No significant association between inner cell mass degree and sex ratio was observed. However, as compared with IVF treatment, all morphology parameters achieved the similar or the biased sex ratio favoring female in ICSI treated subgroup. Quality of blastocysts was positively associated with sex ratio. TE score and expansion degree rather than ICM were significantly associated with sex ratio at birth. ICSI treatment promotes the biased sex ratio favoring female.

## Introduction

The sex ratio at birth is often defined as the proportion of males in all live births^1^. Evolutionary theory suggests that some animal species, including human beings, may adjust their offspring sex ratio in response to maternal health or environmental conditions^2^. Variation in sex ration was commonly observed within human populations, deviating from the equality ratio of males to females (50%)^3–6^. This sex ratio shift was also observed in peoples who underwent assisted reproductive technology (ART) treatment for infertility^7^.

Accumulating evidence suggest that single blastocyst transfer induces a male-biased imbalance in the sex ratio. Sex ratios favoring males have been reported after transfer at the blastocyst stage in in vitro* fertilization* (IVF) cycles^8–10^. On the other hand, it appears that significantly more females were born than expected after blastocyst transfer using ICSI treatment for male infertility^11,12^. As well, a subgroup analysis comparing IVF, intracytoplasmic sperm injection (ICSI) and frozen embryo replacement revealed more females born after ICSI and frozen embryo replacement^9^. However, no difference in sex ration was observed after fresh blastocyst transfer in IVF cycles in another study by Gareth W. et al.^13^. In humans, the sex is determined at the moment of fecundation, while it is actually unclear how single blastocyst transfer in ART induced an imbalance of sex ratio in birth after fecundation. And these conflicting studies suggest that further research is needed in this area with regard to factors possibly affecting the sex ratio.

The selection of human blastocyst for transfer is most often based on morphological criteria and development rate. Blastocyst grading for embryo transfers usually occurs on day 5 of culture, and is composed of three parameters: the cellularity of both the inner cell mass (ICM) and trophectoderm (TE) and the degree of blastocoel expansion^14^. Several studies have attempted to identify the relationship between morphology parameters and sex ratio^15^. Ebner et al. reported that male blastocysts had a 2.53 higher chance of obtaining TE of quality A than female ones^16^. The association of Grade B trophectoderm with a higher sex ratio was also observed in a cohort study of transferring 1210 frozen blastocysts via IVF and ICSI treatments^10^. In a large scale study including 7246 women, who underwent fresh or frozen thawed blastocysts transfer, showed that developmental stage and TE score affected the probability of being a boy^17^. However, the limited samples or mixed groups of IVF and ICSI cycles, or fresh and frozen blastocyst transfer, compromised the power of these conclusions in above studies.

To address the relationship of morphology parameters of blastocyst with sex ratio of birth, this study retrospectively analyzed 6361 singleton frozen-thawed blastocyst transfer cycles from two of the large reproductive centers in Shanghai and Wuhan of China. This study will advanced the understanding of the blastocyst morphology-driven sex allocation in ART.

## Methods

### Patients

The present retrospective study was a multicenter cohort study with retrospectively conducted at the Department of Assisted Reproduction of the Ninth People’s Hospital affiliated with Shanghai Jiao Tong University School of medicine involving 3304 frozen-thaw blastocysts during the period from January 2007 to May 2020 and at the Reproductive Medicine Center of Tongji Hospital affiliated with Huazhong University of Science and Technology involving 3057 frozen-thaw blastocysts during the period from January 2019 to December 2020. The routine ovarian stimulation protocols were applied in 6161 patients in this study. The gonadotropin-releasing hormone (GnRH) agonist long and short protocols, progestin-primed ovarian stimulation (PPOS), gonadotropin-releasing hormone-antagonist (GnRH-A) protocol and micro-stimulus accounted for 36.16%, 30.92%, 24.37%, and 8.55%. The cycles with fertilization of IVF or ICSI followed by frozen-thawed embryo transfer (FET) cycles with single embryo transfer resulting in a single pregnancy and singleton live birth were included. In the case of identical twins, we analyzed only one newborn to ensure independence of observations. Patients lost to follow-up were excluded. Reproductive history includes infertility type –primary and secondary type and cause of infertility-male factor, female factor, both factor and unexplained infertility factor^18^. Female factors mainly include PCOS, tubal factors, uterine disorders, and endometriosis. Male factors mainly include oligospermia and asthenospermia and azoospermia.

### Ethics statement

All methods were performed in accordance with the relevant guidelines and regulations. Approval for human retrospective analysis was obtained from the institutional Ethics Committee of Shanghai Ninth People’s Hospital and Wuhan Tongji Hospital. All participants provided informed consent after counseling for infertility treatments and routine IVF and ICSI procedures.

### Patient and public involvement

This study involved the existing data and not included patients as study participants. There were no patients involved in setting the research question, the study design or the overall conduct of this study. No plans were involved patients in the dissemination of study findings.

### Laboratory protocols

Oocytes were retrieved approximately 36 h after HCG administration. All the aspirated oocytes were fertilized with conventional insemination (IVF) or intracytoplasmic sperm injection (ICSI). Fertilization was assessed according to the presence of the pronuclear after 16–18 h insemination. One or two best-quality cleavage embryos for fresh cycle were choosed to transfer or cryopreservation 5 or 6 best-quality cleavage embryos according to the first cycle or not. The surplus embryos will be cultured continued and then the blastocyst were vitrified cryopreservation and thawed in FET cycles. In our study, we only analyzed the frozen-thawed blastocyst transfer. The cryoprotectant solution (KITAZATO^@^ Vitrofaction KIT, Japan) consisted of 15% dimethyl sulfoxide, 15% ethylene glycol and 0.5 M sucrose. We usually used laser objective and drill the zona pellucida through 2–3 laser pulses, then vitrified collapsed blastocysts within 30 min to prevent their re-expansion. When thawing blastocysts, the cryoprotectant dilutions (KITAZATO@ 129 Vitrofaction KIT, Japan) were sequential 1, 0.5, 0 M sucrose solutions. All the steps were carried out at room temperature except the first warming step (37 °C).

On day 5, 6, 7 of in vitro cultivation, blastocysts were evaluated morphologically according to Gardner and Schoolcraft´s classification^19^. The time of embryos observation is usually between 8 and 10 am. Blastocysts were assigned 3, 4, 5, 6 numeric score based on the degree of expansion and hatching status and ICM (A, B, C) and TE (A, B, C) evaluation was based on cell growth^20,21^.

In this study, blastocysts were classified into three groups: “good”, 3-6AA, 3-6AB, 3-6BA; “fair”, 3-6BB, 3-6AC, 3-6CA; “poor”, 3-6BC, 3-6CB, 3-6CC^21–23^. All embryos were cultured at 37 ° with 5% O_2_ and 6% CO_2_ concentration.

### Embryo transfer and clinical outcomes

FET was performed after via a natural cycle or an artificial cycle. Women who have regular menstrual cycles underwent a natural cycle FET and women with irregular cycles an artificial cycle was offered with sequential oral administration of estradiol valerate and micronized vaginal progesterone as previously described^24^. The cycles with fertilization of IVF or ICSI followed by frozen-thawed embryo transfer (FET) cycles with single embryo transfer resulting in a single pregnancy and singleton live birth were included. In our study, the primary outcome measure was sex ratio which was calculated as the proportion of male newborns among all live births.

### Statistical analysis

We performed matching using propensity scores calculated using logistic regression model in SPSS version 25.0. The inclusion criteria for good-fair-poor blastocyst groups was matched one-to-one on maternal age, maternal BMI, paternal age, duration of infertility, basal FSH, basal LH, type of infertility, main infertility cause (female factor, male factor, mixed factor and unexplained infertility), type of fertilization, day of embryo transfer which was found to potentially affect the sex ratio or significant different between these three groups. 791 of 1354 FET cycles of good blastocyst, 791 of 3592 FET cycles of fair blastocyst, 791 of 1415 FET cycles of poor blastocyst were obtained using nearest neighbor matching, with a caliper width of 0.02 and without replacement. All continuous data were presented as the mean and standard deviation (SD). Categorical variables were expressed as percentages and were analyzed using the chi-square test or Fisher’s exact test. A Binary logistic regression analysis was used to assess the association between the sex ratio of live birth and various morphological parameters. The data were reported as odds ratios (OR) and 95% confidence intervals (95% CI). A value of P < 0.05 was referred to as a statistical significance.

### Ethics approval and consent to participate

Approval for human retrospective analysis was obtained from the institutional Ethics Committee of Shanghai Ninth People’s Hospital and Wuhan Tongji Hospital. All participants provided informed consent after counseling for infertility treatments and routine IVF and ICSI procedures.

## Results

### Demographics and basic characteristics

A total of 6361 singleton delivery offspring after frozen-thawed blastocyst transfer from two of the large reproductive centers in Shanghai and Wuhan of China were included in the cohort. 1354 good blastocyst, 3592 fair blastocyst and 1415 poor blastocyst were included. No significance were found in type of infertility, main infertility cause between these three groups (Supplementary Table 1). Also no significance were found in the rates of maternal age, paternal age, duration of infertility between good and fair blastocyst. However we found that there were significant differences in maternal age a, maternal BMI and paternal age between Good and Poor or Fair and Poor blastocysts. And Good, Fair and Poor blastocyst were significantly higher in IVF group than ICSI group. Also Day of embryo transfer was significant differences between these three groups (Supplementary Table 1). These differences may have effects on sex ratio outcomes, so we performed the propensity score matching to control for confounding parameters. After matching, there were 791 Good, 791 Fair and 791 Poor blastocysts in our data and the basic characteristic of these three groups were similar (P > 0.05, Table 1).

**Table 1:** The basic characteristic of patients udergoing ART treatment between the groups of good-fair-poor blastocysts after matching.

Patients characteristic	Good blastocyst	Fair blastocyst	Poor blastocyst	P1	P2	P3
Number of cycles	791	791	791			
Mean maternal age (years ± SD)	34.02 ± 5.26	33.92 ± 5.02	33.84 ± 4.87	0.718^a^	0.485^a^	0.734^a^
Maternal BMI (kg/m^2^)	21.62 ± 2.94	21.76 ± 2.93	21.63 ± 3.01	0.330^a^	0.942^a^	0.374^a^
Mean paternal age (years ± SD)	35.85 ± 6.05	35.83 ± 5.80	35.73 ± 5.79	0.922^a^	0.668^a^	0.735^a^
Mean duration of infertility (years ± SD)	3.01 ± 2.21	2.91 ± 2.28	2.88 ± 2.37	0.354^a^	0.265^a^	0.839^a^
Basal FSH	6.73 ± 2.02	6.69 ± 1.79	6.68 ± 1.84	0.669^a^	0.553^a^	0.855^a^
Basal LH	5.22 ± 3.45	5.01 ± 3.29	5.09 ± 3.52	0.200^a^	0.451^a^	0.619^a^
Type of infertility (%)						
Primary	479 (60.6%)	460 (58.2%)	457 (57.8%)	0.480^b^		
Secondary	312 (39.4%)	331 (41.8%)	334 (42.2%)			
Main infertility cause (%)						
Female factor	505 (63.84%)	506(63.97%)	505(63.84%)	1.000^b^		
Male Factor	103 (13.02%)	100 (12.64%)	102 (12.90%)			
Mixed factor	134 (16.94%)	135 (17.07%)	134 (16.94%)			
Unexplained infertility	49 (6.20%)	50 (6.32%)	50 (6.32%)			
Type of fertilization (%)						
IVF	516 (65.23%)	532 (67.26%)	537 (67.89%)	0.512^b^		
ICSI	275 (34.77%)	259 (32.74%)	254 (32.11%)			
Day of embryo transfer (%)						
D5	480 (60.68%)	471 (59.55%)	480 (60.68%)	0.987^b^		
D6	304 (38.43%)	312 (39.44%)	304 (38.43%)			
D7	7 (0.89%)	8 (10.11%)	7 (0.89%)			

The sex ratio was defined as the porportion of males in all live births.

*BMI* body mass index, *IVF* in vitro fertilization, *ICSI* intracytoplasmic sperm injection.

P1: Good blastocyst group versus Fair blastocyst group. P2: Good blastocyst group versus Poor blastocyst group. P3: Fair blastocyst group versus Poor blastocyst group.

^a^One-way ANOVA. Values are mean ± SD.

^b^Pearson chi-square test. Values are number (percentage).

It was found that the sex ratio was significantly higher in IVF group than that of ICSI group before and after matching (56.6% versus 51.9%, P < 0.001; 57.4% versus 51.3%, P < 0.01, Supplementary Fig. 1A). Both Good and Poor quality blastocysts in IVF group resulted in a significant higher sex ratio than those in ICSI group before and after matching (P < 0.001, P < 0.05; P < 0.05, P < 0.05, respectively, Supplementary Fig. 1C). In addition, no difference was observed in birth weight between ICSI and IVF treated groups (Supplementary Fig. 1B). So we have reason to think that the matched data can be analyzed on behalf of all the data.

### Association between blastocyst morphology parameters and sex ratio

We compared the possible influence of blastocyst quality on sex ratio. Transplantation of Good quality blastocyst resulted in a significantly higher sex ratio than those of poor-quality blastocyst (58.15% versus 51.71%, P = 0.011, odds ratio [OR], 1.295; 95% CI 1.062–1.581).

Since TE, ICM and degree of blastocyst expansion are kinetic parameters in morphological grades, we next explored their reference values for sex ratio. There was no significant difference among ICM grades with regard to sex ratio (Table 2). However, the group with A grade of TE achieved a much higher sex ratio of 61.68%, while the group with C grade of TE achieved a lower sex ratio of 49.35% (P < 0.001; odds ratio [OR], 1.653; 95% CI 1.281–2.134, Table 2). As well, the group with B grade of TE achieved higher sex ratio of 56.66% than that of C grade of TE (P = 0.003; odds ratio [OR], 1.322; 95% CI 1.098–1.593, Table 2). Moreover, blastocysts transferred with an expansion degree 6 had a 29.53% higher probability of sex ratio than those with an expansion degree 4 + 3 (P < 0.001; odds ratio [OR], 4.406; 95% CI 1.957–9.919, Table 2). Although, there was a decrease of sex ratio in the group of the expansion degree 5, no statistical differences were observed as compared with the group of expansion degree 4 + 3 (50.65% versus 54.91%, P = 0.1000.510, Table 2). We also found that there were no differences in sex ratio when transfer frozen-thawed blastocysts of day 5, day 6 or day 7 (55.07% versus 55.98% versus 45.45%, P = 0.935, P = 0.325, Table 2).

**Table 2:** Association between blastocyst morphology parameters and sex ratio.

Morphological parameter	Grade	Singletons (n)	Male (n)	Sex ratio (%)	P	OR	95% CI
Blastocyst	Good	791	460	58.15	0.011	1.295	1.062–1.581
	Fair	791	444	56.13	0.082	1.193	0.978–1.454
	Poor	791	409	51.71	Ref		
ICM grade	A	687	393	57.21	0.221	0.770	0.490–1.125
	B	1574	848	53.88	0.059	0.678	0.453–1.014
	C	112	72	64.29	Ref		
TE grade	A	381	235	61.68	0.000	1.653	1.281–2.134
	B	1299	736	56.66	0.003	1.322	1.098–1.593
	C	693	342	49.35	Ref		
Expansion degree	6	45	38	84.44	0.000	4.406	1.957–9.919
	5	77	39	50.65	0.510	0.858	0.544–1.354
	4 + 3	2251	1236	54.91	Ref		
Transfer day	7	22	10	45.45	0.935	1.007	0.846–1.199
	6	920	515	55.98	0.325	0.65	0.275–1.533
	5	1431	788	55.07	Ref		

*OR* Odds ratio, *CI* Confidence interval, *TE* Trophectoderm, *ICM* Inner cell mass, *ref* reference.

### Effect of insemination type on sex ratio

To assess the effect of insemination type on sex ratio, blastocysts were allocated to IVF or ICSI derived group. It was found that the sex ratio was significantly higher in IVF group than that of ICSI group (Supplementary Fig. 1A). Both in IVF group and ICSI group, the blastocyst quality was significantly positive with the sex ratio (P < 0.05, Table 3). TE grades exhibited a strong effect on sex ratio likewise in both two groups (IVF group: 63.18% versus 58.62% versus 52.16%, P < 0.05; and ICSI group: 59.15% versus 52.52% versus 43.67%, P < 0.05, Table 3). Interestingly, blastocysts with expansion degree 6 in IVF group achieved a higher sex ratio (P = 0.001; odds ratio [OR], 4.957; 95% CI 1.919–12.805, Table 3), while in ICSI group, although blastocysts with expansion degree 6 has higher sex ratio than expansion degree 5 than expansion degree 4 + 3, there was no significantly differences between these three groups. In subgroup comparison, transferring blastocysts of day 6 had the highest sex ratio both in IVF and ICSI groups (57.48%, 52.63%, respectively, Table 3).

**Table 3:** Effect of insemination type on sex ratio.

Morphological parameter	Grade	IVF					ICSI				
		Singletons (n)	Sex ratio (%)	P	OR	95% CI	Singletons (n)	Sex ratio (%)	P	OR	95% CI
Blastocyst	Good	516	61.24	0.031	1.312	1.025–1.679	275	52.36	0.145	1.291	0.915–1.821
	Fair	532	56.77	0.436	1.101	0.864–1.403	259	54.83	0.047	1.423	1.005–2.016
	Poor	537	54.38	Ref			254	46.06	Ref		
ICM grade	A	454	60.13	0.357	0.792	0.482–1.301	233	51.50	0.373	0.698	0.316–1.540
	B	1049	55.58	0.099	0.668	0.414–1.078	525	50.48	0.309	0.672	0.313–1.444
	C	82	65.85	Ref			30	60.00	Ref		
TE grade	A	239	63.18	0.005	1.578	1.144–2.177	142	59.15	0.004	1.889	1.232–2.897
	B	882	58.62	0.034	1.280	1.019–1.608	417	52.52	0.033	1.426	1.029–1.975
	C	464	52.16	Ref			229	43.67	Ref		
Expansion degree	6	38	86.84	0.001	4.957	1.919–12.805	7	71.43	0.290	2.442	0.468–12.748
	5	68	51.47	0.452	0.829	0.508–1.352	9	44.44	0.698	0.769	0.204–2.894
	4 + 3	1479	56.93	Ref			772	51.04	Ref		
Transfer day	7	12	50.00	0.595	1.369	0.430–4.354	10	40.00	0.448	1.658	0.449–6.129
	6	635	57.48	0.642	1.317	0.413–4.194	285	52.63	0.375	1.807	0.489–6.687
	5	938	57.46	Ref			493	50.51	Ref		

*OR* Odds ratio, *CI* Confidence interval, *TE* Trophectoderm, *ICM* Inner cell mass, *ref* reference.

### Interaction of insemination type and blastocyst morphology on sex ratio

**Figure 1:**
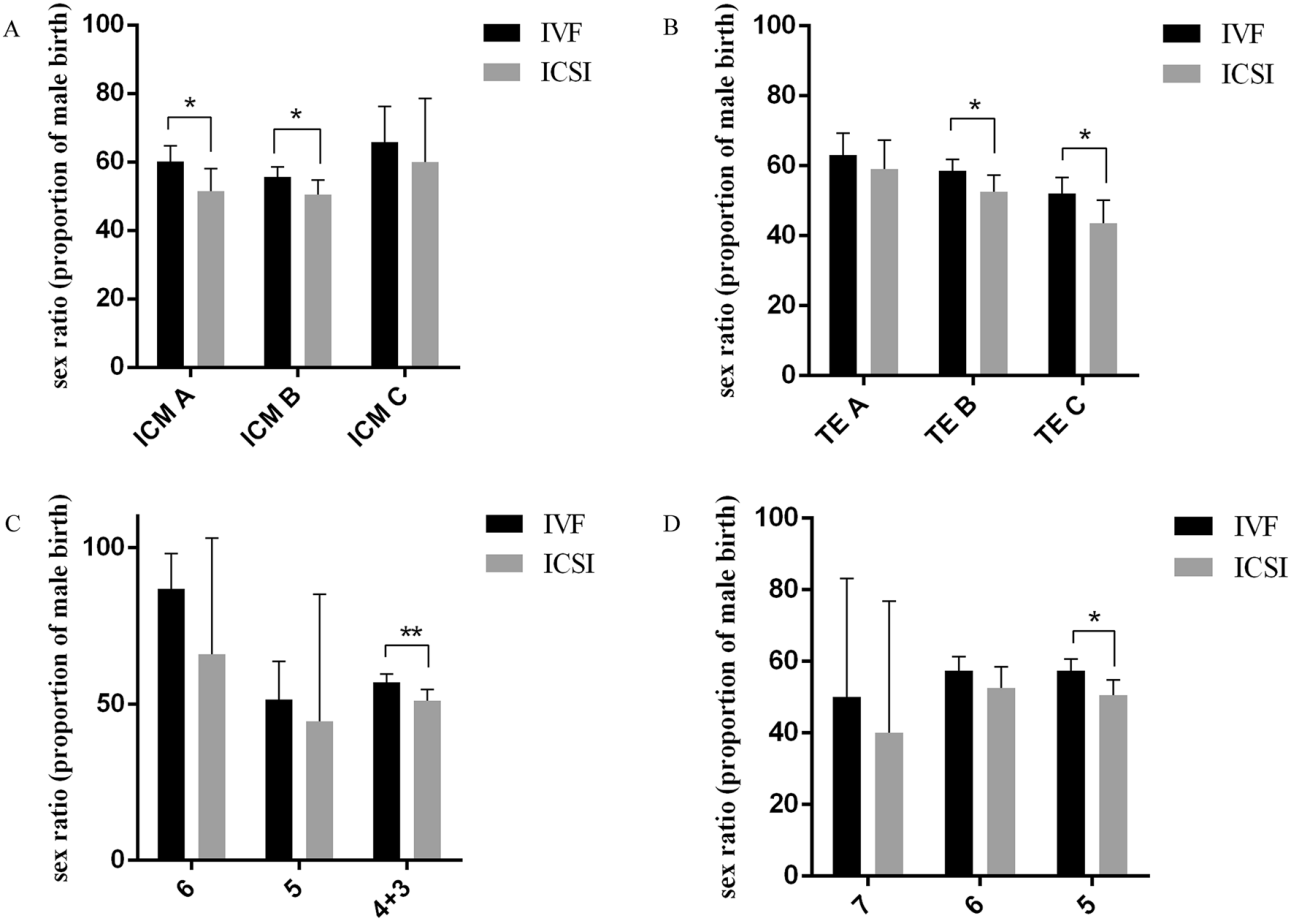
Interaction of insemination type and blastocyst morphology on sex ratio. (**A**) Effects of ICM grade on sex ratio in IVF and ICIS treated groups. (**B**) Effects of TE grade on sex ratio in IVF and ICIS treated groups. (**C**) Effects of blastocysts expansion grade on sex ratio in IVF and ICIS treated groups. (**D**) Effects of blastocysts transfer day on sex ratio in IVF and ICIS treated groups. Data were presented with ratio with 95% confidence intervals (CIs). **P < 0.01, *P < 0.05, chi-square test.

To further explore the interaction of insemination type and blastocyst morphology on sex allocation, stratification analyses was performed and showed that good-quality and poor-quality blastocysts in IVF group resulted in a significant higher sex ratio than those in ICSI group (Supplementary Fig. 1C). Furthermore, there was a significant increase in sex ratio of IVF group both in A and B grade of ICM blastocysts as compared with ICSI group (ICM A: 60.13% versus 51.50%, P = 0.03; ICM B: 55.58% versus 50.48%, P = 0.031, Fig. 1A). In the perspective of TE grade, IVF-derived blastocysts with the B and C grade TE achieved higher sex ratio than those in ICSI group (58.62% versus 52.52%, P = 0.038; 52.16% versus 43.67%, P = 0.036, respectively, Fig. 1B). In addition, a higher sex ratio was observed in IVF-derived blastocysts with the expansion degree of 4 + 3 as compared with ICSI group (56.93% versus 51.04%, P = 0.008, Fig. 1C). Also, a 6.95% higher sex ratio in transferring blastocysts of day 5 in IVF group than those in ICSI group (57.46% versus 50.51%, P = 0.012, Fig. 1D).

## Discussion

To our knowledge, this is the largest multicenter cohort study assessing the potential associations between morphology parameters of the frozen-thawed blastocysts and the sex ratio of singleton delivery offspring. We found that blastocyst quality, TE score, and expansion degree were associated with sex ratio both in IVF and ICSI groups, while no association was observed for ICM score and sex ratio. The major strength of this study is that we presented the sex allocation of a high number of competent blastocysts leading to a live birth from two big reproductive centers in China. The high number of outcome data led to relatively narrow CIs and therefore high precision of the results.

Although the studies about the association between blastocyst transfer and sex ratio published so far, some results are conflicting^8,13,25–28^. Here, our data showed that blastocyst transfer preferred male birth with 9.8% higher than female birth. It was reported that the female embryos with more active metabolism may be under oxidative stress^29,30^, which might make them more liable for growth retardation and poor quality than male embryos. Although high glucose metabolism was also observed in the male embryo from morula to blastocyst both in humans and cows^31,32^, these male embryos could develop to robust blastocysts probably due to being less susceptible to oxidative stress^33^. Therefore, more male blastocysts may be selected for fresh or frozen-thaw embryo transfer cycles, leading to a bias sex ratio.

Based on the morphology grading, competent blastocysts are selected for transfer at our IVF centers. To achieve satisfying pregnancies, those blastocysts showing complete expansion of the blastocoel cavity and hatching potential with good numbers of cells in both the TE and ICM were preferred for transfer. As a consequence, transfer of blastocysts with high quality contributed to more male birth in our study as well as reported previously^8^. Borgstrom et al. conducted a cohort study of 4842 deliver offspring from fresh or frozen-thawed embryo transfer and found that TE A blastocysts as compared with TE B blastocysts had a 31% increased probability of being a boy^17^. We also observed a significant increasing trend in the sex ratio in the context of TE A and B blastocysts transfer as compared with TE C blastocysts transfer both in ICSI and IVF groups, suggesting that high TE quality may increase the proportion of male infants. Similarly, blastocysts with expansion degree 6 owned a higher sex ratio, which is consistent in comparison of mixed group of IVF and ICSI derived blastocysts or IVF derived blastocysts alone. However, the ICSI derived blastocysts transfer with expansion degree 5 rather than 6 showed a higher sex ratio, which might be relative with the small number of samples in this subgroup. Interestingly, no statistically significant associations between ICM scores and sex ratio were found both in IVF and ICSI groups, although ICM was an important sign for high quality blastocysts.

It was reported that insemination type also affect sex ratio^34–37^. Here, we found that fewer male live-born infants are conceived via ICSI than IVF just as reported previously^9,38,39^. ICSI is the method of choice in cases with reduced sperm activity. In general, normal sperm with morphologically good are preferentially selected under high magnification for ICSI and thus induced female-biased births, since the percentage of sperm with good morphology carrying an X chromosome is higher^39^. Compared with IVF treated group, both the overall quality grading and the individual parameter score, including ICM, TE, and expansion degree, promoted the bias of sex ratio favoring female in ICSI treated group. These results indicated that the balance of sex ratio might be affected by insemination procedure and blastocyst morphology simultaneously or interactively.

As a large multicenter study, the limitation of the current study comes from the incomplete parent characteristic information for the different electrical data system. In addition, about half of the thawed blastocysts (from the center of Shanghai) were derived from the non-top quality cleavage embryos due to the freeze-all or fresh transfer strategy for all top quality embryos on day 3. It should be considered that the blastocysts with good quality would be higher if all embryos were cultured for blastocyst. As a consequence, the sex ratio favoring male would be higher possibly.

### Perspective and significance

This large multicenter cohort study expands the current knowledge on associations between blastocyst morphology parameters and sex allocation of singleton delivery offspring. We demonstrated that the higher quality blastocysts resulted in a higher sex ratio after single frozen-thawed blastocyst transfer. TE score and expansion degree of blastocysts were significantly associated with sex ratio at birth, while ICM score showed no association with sex ratio whatever in ICSI or IVF treated groups. However, as compared with IVF treatment, all morphology parameters promoted the sex ratio bias favoring female in ICSI treated group, indicating the interaction of insemination type and blastocyst morphology on sex allocation. Embryologists should be aware of the effects of certain protocols on sex distribution, including blastocyst transfer and insemination method, in order to maintain the equilibrium of the sex ratio among offspring without affecting the pregnancy rate.

### Supplementary Information

Supplementary Information.

## Data Availability

The data used for this study comes from department of assisted reproduction of Shanghai Ninth People’s hospital and Tongji Hospital. Please contact corresponding author for data requests.
