# Supplementary material for: Sex ratio shift after frozen single blastocyst transfer in relation to blastocyst morphology parameters
Source: Sci Rep. 2024 Apr 25;14:9539. doi: 10.1038/s41598-024-59939-y (PMC11045847; doi:10.1038/s41598-024-59939-y)
Supplement: Supplementary file 1 — Supplementary Information. [file 41598_2024_59939_MOESM1_ESM.pdf]

Supplementary Table 1 Demographics and basic characteristic of patients undergoing ART treatment before matching.

**Patients characteristic**	**Good blastocyst**	**Fair blastocyst**	**Poor blastocyst**	**P1**	**P2**	**P3**
Number of cycles	1354	3592	1415			
Mean maternal age (years ±SD)	33.62±5.33	33.75±4.97	34.56±5.05	0.434[a]	0.000[a]	0.000[a]
Maternal BMI (kg/m²)	21.56±2.85	21.75±3.08	21.80±3.09	0.042[a]	0.036[a]	0.618[a]
Mean paternal age (years ±SD)	35.53±6.09	35.66±5.89	36.39±5.88	0.492[a]	0.000[a]	0.000[a]
Mean duration of infertility (years ±SD)	2.94±2.19	2.99±2.32	3.07±2.46	0.528[a]	0.165[a]	0.304[a]
Basal FSH	6.74±1.95	6.76±1.96	6.79±2.09	0.756[a]	0.470[a]	0.576[a]
Basal LH	5.41±3.81	5.21±3.96	4.96±3.39	0.101[a]	0.001[a]	0.030[a]
Type of infertility (%)						
Primary	796(58.79%)	2155(60.00%)	835(59.01%)	0.672[b]		
Secondary	558(41.21%)	1437(40.00%)	580(40.99)			
Main infertility cause(%)						
Female factor	866(63.96%)	2300(64.03%)	922(65.16%)	0.980[b]		
Male Factor	185(13.66%)	484(13.47%)	177(12.51%)			
Mixed factor	23517.36%)	629(17.51%)	245(17.31%)			
Unexplained infertility	68(5.02%)	179(4.98%)	71(5.02%)			
Type of fertilization (%)						
IVF	907(66.99%)	2344(65.26%)	869(61.41%)	0.006[b]		
ICSI	447(33.01%)	1248(34.74%)	546(38.59%)			

Day of embryo transfer (%)				
D5	1041(76.88%)	2028(56.46%)	481(33.99%)	0.000[b]
D6	306(22.60%)	1513(42.12%)	903(63.82%)	
D7	7(0.52%)	51(1.42%)	31(2.19%)	

Note: The sex ratio was defined as the proportion of males in all live births. BMI=body mass index; IVF=in vitro fertilization; ICSI=intracytoplasmic sperm injection. P1: Good blastocyst group versus Fair blastocyst group. P2: Good blastocyst group versus Poor blastocyst group. P3: Fair blastocyst group versus Poor blastocyst group.

[a]One-way ANOVA. Values are mean ± SD.

[b]Pearson chi-square test. Values are number (percentage).

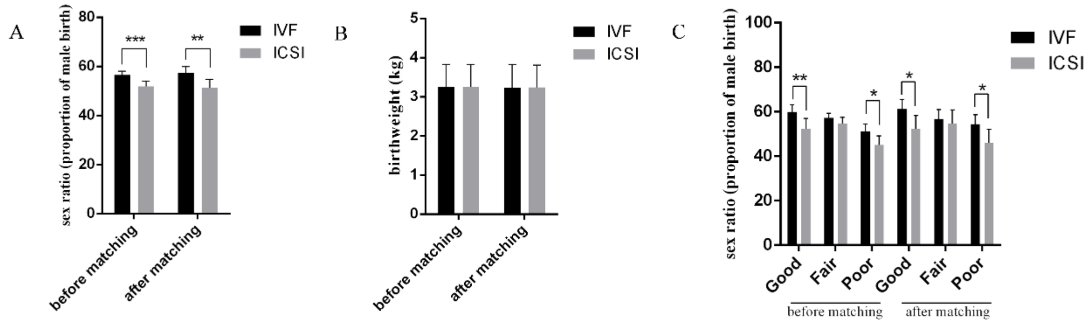

Supplemental Figure 1 Effects of insemination type and blastocyst morphology on sex ratio. A) Effects of ICM Grade on sex ratio between IVF and ICIS treated groups. B) The association between insemination and birthweight. C) Effects of blastocysts quality on sex ratio in IVF and ICIS treated groups. Data were presented with ratio with 95% confidence intervals (CIs) in (A) and (C) and mean value ± SD in (B). ***P<0.001, **P<0.01, *P<0.05, chi-square test.
